# YouGenMap: a web platform for dynamic multi-comparative mapping and visualization of genetic maps

**DOI:** 10.3389/fgene.2014.00183

**Published:** 2014-06-24

**Authors:** Keith Batesole, Kokulapalan Wimalanathan, Lin Liu, Fan Zhang, Craig S. Echt, Chun Liang

**Affiliations:** ^1^Department of Computer Science and Software Engineering, Miami UniversityOxford, OH, USA; ^2^Department of Biology, Miami UniversityOxford, OH, USA; ^3^Southern Research Station, Southern Institute of Forest Genetics, USDA Forest ServiceSaucier, MS, USA

**Keywords:** YouGenMap, genetic map, genetic map viewer, genetic marker, graphic user interface

## Abstract

Comparative genetic maps are used in examination of genome organization, detection of conserved gene order, and exploration of marker order variations. YouGenMap is an open-source web tool that offers dynamic comparative mapping capability of users' own genetic mapping between 2 or more map sets. Users' genetic map data and optional gene annotations are uploaded, either publically or privately, as long as they follow our template which is available in several standard file formats. Data is parsed and loaded into MySQL relational database to be displayed and compared against users' genetic maps or other public data available on YouGenMap. With the highly interactive GUIs, all public data on YouGenMap are maps available for visualization, comparison, search, filtration and download. YouGenMap web tool is available on the website (http://conifergdb.miamioh.edu/yougenmap) with the source-code repository at (http://sourceforge.net/projects/yougenmap/?source=directory).

## Introduction

Genetic linkage maps, also known as genetic maps can be used to determine the order of genes on chromosomes and how genetic markers are arranged and the approximate distances among them. The rapid accumulation of genomics data and genome sequences has allowed rapid development of many SNPs (Single Nucleotide Polymorphism), SSRs (Simple Sequence Repeat) and other markers. Consequently, there are growing demands for bioinformatics tools that can be easily picked up and utilized by biologists to examine, visualize, compare, consolidate, and update linkage maps. More so, comparative genetic mapping between species or within species allows examination of genome organization, detection of conserved gene order between species, and exploration of marker order variations among pedigrees or mapping methods (Baxter et al., 2011; Khan et al., 2012; Pavy et al., 2012).

The GMOD open-source tool—CMAP (Youens-Clark et al., 2009) has been widely utilized by the research community for comparative genetic map visualization. Unfortunately, it is limited to the comparison of two adjacent aligned maps for correspondence and requires constant page navigation. As a Perl-CGI application using a relational database, CMAP runs on an Apache web server and does not allow users to upload and integrate their own marker data using web browsers. Implemented using basic HTML/JavaScript, CMAP's web interfaces offer very limited user interaction. While NCBI's MapViewer (Wolfsberg, 2010) can compare multiple maps, it can be used only for markers and sequence data curated by NCBI. MapChart (Voorrips, 2002) and Circos (Krzywinski et al., 2009) have been used for comparative map visualization (Echt et al., 2011; Lucas et al., 2011; Pavy et al., 2012), but do not provide for an interactive web platform. To overcome limitations of extant map comparison and visualization tools, YouGenMap was developed.

## Implementation

YouGenMap is an open-source web tool implemented using a JavaScript/HTML frontend and a PHP/MySQL backend. It was developed through a combination and collaboration of various JavaScript libraries and PHP libraries. Dojo (http://dojotoolkit.org), a JavaScript library, was used to provide rich GUI elements and dynamic interaction with the server's file directory and relational database through AJAX. RaphaelJS (http://raphaeljs.com), a JavaScript library, was used to provide dynamic interaction with the SVG (Scalable Vector Graphics) elements for creating and displaying genetic maps. PHPExcel (http://phpexcel.codeplex.com), a PHP library, was used to create, modify, and parse spreadsheet files such as Microsoft Excel and Open Office Documents. ADOdb (http://adodb.sourceforge.net/), a PHP library, was used to provide a consistent API for accessing a variety of databases such as MySQL and Oracle in the same way. ImageMagick (http://www.imagemagick.org), a PHP library, was used to convert a SVG file to a PNG file. YouGenMap is designed to be compatible in all major web browsers including Google Chrome, Mozilla Firefox, and Microsoft Internet Explorer (9.0 and above) across different OS platforms.

## Web interface and usage

YouGenMap is a genetic map viewer that lets users upload, download, display, visualize, update and compare sets of mapping and marker annotation data. Users' genetic map data is uploaded and downloaded as a spreadsheet using the map set template we provide. The map set template file can be one of four formats: Microsoft Workbook, Microsoft 1997–2003 Worksheet, Open Document Format (Open Office/LibreOffice), or the Microsoft XML Spreadsheet. Upon an upload, the map set template file is parsed and placed into our relational database. Our map set template file, both in simple version (i.e., Supplemental Files No.1 and No.3) and complete version (i.e., Supplemental Files No.2 and No.4), contains full instructions on how to modify the map set file to incorporate users' data that meets our data formatting requirements for uploading and processing.

**Figure 1:**
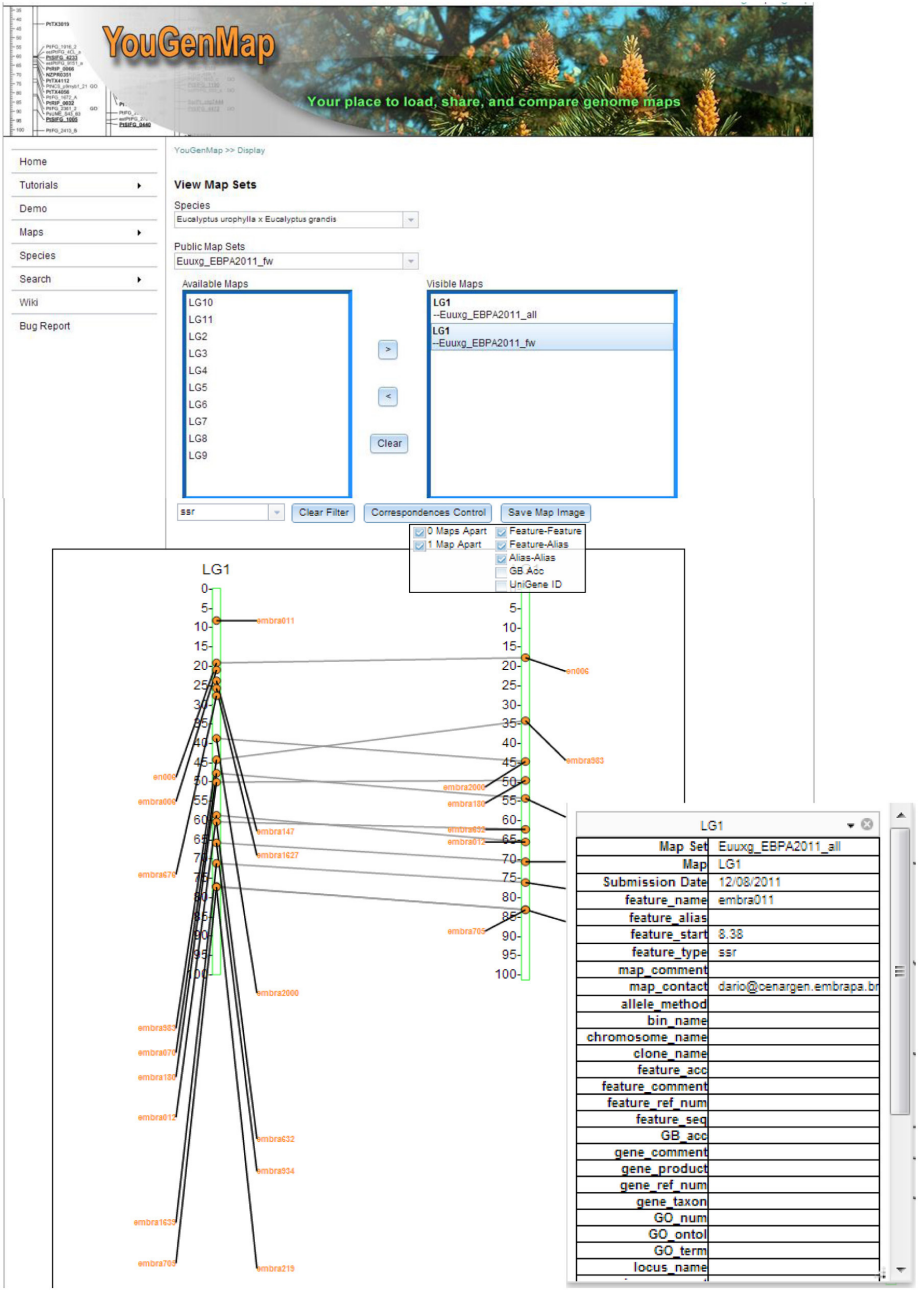
**A snap-shot of YouGenMap web interface that shows comparative mapping**.

As shown in Figure 1, YouGenMap allows users to visualize multiple map sets at a time and has flexible options for displaying correspondences among maps, which currently include nine types of marker (feature) sequence and gene identifiers. The correspondence lines between markers can be drawn between two maps that are not adjacent (e.g., between two maps with a map in between them). A user can selectively display desirable features by applying a filter on a feature type and the type of correspondence (e.g., features, aliases, UniGene ID, etc). A user can also flip a map and be able to take a snapshot of a current comparison (saved as a PNG image). Moreover, clicking a feature in a displayed map will show its annotations (e.g., GO term, GO number, and UniGene and reference protein information) and map data in details. However, annotation information has to be provided by users and is available only if the complex version of map set template file is utilized for data input. Users can easily register and create their own accounts on our website and have the option to make their data public or private accessible. Any public maps can be compared against a user's own maps as well as downloaded. YouGenMap's correspondence drawing capabilities provides a powerful tool for comparative mapping.

## Conclusion

While a few map drawing applications and web tools can be used for comparative mapping, they are either platform-specific (i.e., MapChart Voorrips, 2002) or require intervention by a database manager (i.e., CMap Youens-Clark et al., 2009 and NCBI Map Viewer Wolfsberg, 2010). In contrast, easy-to-use and a highly interactive web interface are two major characteristics of YouGenMap. With YouGenMap, genetic maps and their annotations could become dynamic community assets. So far, we have hosted 10 map sets for 5 tree species. Also a public bug tracking system and tutorial videos/documents are available online. As an open source took, we are in the process of improving its functionality to better serve the research community.

### Conflict of interest statement

The authors declare that the research was conducted in the absence of any commercial or financial relationships that could be construed as a potential conflict of interest.

## Supplementary material

Supplemental File No.1 **Simplified sample data template in Excel that a user can use to store his/her own data and upload this data into YouGenMap web server**.

Supplemental File No.2 **Complete sample data template in Excel that a user can use to store his/her own data and upload this data into YouGenMap web server**.

Supplemental File No.3 **Simplified sample data template in OpenDocument Spreadsheet that a user can use to store his/her own data and upload this data into YouGenMap web server**.

Supplemental File No.4 **Complete sample data template in OpenDocument Spreadsheet that a user can use to store his/her own data and upload this data into YouGenMap web server**.
